# Distribution and prevalence of Sin Nombre hantavirus in rodent species in eastern New Mexico

**DOI:** 10.1371/journal.pone.0296718

**Published:** 2024-01-18

**Authors:** Jaecy K. Banther-McConnell, Thanchira Suriyamongkol, Samuel M. Goodfellow, Robert A. Nofchissey, Steven B. Bradfute, Ivana Mali

**Affiliations:** 1 Department of Biology, Eastern New Mexico University, Portales, New Mexico, United States of America; 2 College of Agricultural Sciences, Southern Illinois University-Carbondale, Carbondale, Illinois, United States of America; 3 Center for Global Health, Department of Internal Medicine, University of New Mexico Health Sciences Center, Albuquerque, New Mexico, United States of America; 4 Fisheries, Wildlife, and Conservation Biology Program, North Carolina State University, Raleigh, North Carolina, United States of America; University of Minnesota, UNITED STATES

## Abstract

Orthohantaviruses are diverse zoonotic RNA viruses. Small mammals, such as mice and rats are common chronic, asymptomatic hosts that transmit the virus through their feces and urine. In North America, hantavirus infection primarily causes hantavirus cardiopulmonary syndrome (HCPS), which has a mortality rate of nearly 36%. In the United States of America, New Mexico (NM) is leading the nation in the number of HCPS-reported cases (N = 129). However, no reported cases of HCPS have occurred within eastern NM. In this study, we assessed the prevalence of Sin Nombre virus (SNV) in rodent assemblages across eastern NM, using RT-qPCR. We screened for potential rodent hosts in the region, as well as identified areas that may pose significant infection risk to humans. We captured and collected blood and lung tissues from 738 rodents belonging to 23 species. 167 individuals from 16 different species were positive for SNV RNA by RT-qPCR, including 6 species unreported in the literature: *Onychomys leucogaster* (Northern grasshopper mouse), *Dipodomys merriami* (Merriam’s kangaroo rat), *Dipodomys ordii* (Ord’s kangaroo rat), *Dipodomys spectabilis* (Banner-tailed kangaroo rat), *Perognathus flavus* (Silky pocket mouse), and *Chaetodipus hispidus* (Hispid pocket mouse). The infection rates did not differ between sexes or rodent families (i.e., Cricetidae vs. Heteromyidae). Generalized linear model showed that disturbed habitat types positively influenced the prevalence of SNV at sites of survey. Overall, the results of this study indicate that many rodent species in east New Mexico have the potential to maintain SNV in the environment, but further research is needed to assess species specific infectivity mechanisms and potential risk to humans.

## Introduction

Orthohantaviruses (hantaviruses) are widely distributed and diverse zoonotic RNA viruses with over 20 species causing diseases in humans [1, 2]. Many small mammals including rodents (i.e., mice, rats, voles) are asymptomatic viral hosts that can shed virions in feces, urine, and saliva and an infection may occur upon inhaling aerosolized viral particles or through deposits of infected saliva into fresh wounds [1, 3–7]. In these host species, several organs can host viral loads such as lung, heart, liver, kidney, and, in lower quantities, the spleen [8–11]. Hantaviruses are broadly classified into two categories respective of their regions of origin and diseases they can cause in humans: Old World hantaviruses target renal system and can cause hemorrhagic fever with renal syndrome (HFRS) and New World hantaviruses target cardiovascular system and can cause hantavirus cardiopulmonary syndrome (HCPS) [1, 12–14]. Most rodent-to-human infections can be attributed to rodent reservoir hosts within the Muridae and Cricetidae families [15, 16]. Human activities that disturb the environment in which infected mice may have nested, occupied, or excreted waste in for a long period of time poses risk of hantavirus infection [1].

The history of hantavirus disease in North America began with the 1993 outbreak of a fatal respiratory illness in the Four-Corners region of the United States of America (USA), where the boundaries of New Mexico, Arizona, Utah, and Colorado meet [17–20]. Shortly after, a new strain of hantavirus was isolated from the tissue of North American deer mouse, *Peromyscus maniculatus*, and named Sin Nombre virus (SNV) [17–20]. SNV is thought to be responsible for most North American cases of HCPS, with *P*. *maniculatus* serving as the primary reservoir host [17–20]. Since *P*. *maniculatus* is a habitat generalist and known to opportunistically invade recently disturbed habitats [21], SNV surveillance efforts were traditionally focused on *P*. *maniculatus* and less attention was given to other rodent species and their potential to serve as hantavirus hosts [18, 22, 23].

Over the years, SNV surveillance efforts have included larger rodent assemblages. As a result, SNV genetic material (i.e., RNA) has been detected in the lungs of additional North American Cricetids [18, 24–26]. The overall prevalence of hantavirus in rodent assemblages is thought to be driven by interactions among competent and non-competent hosts (i.e., hosts that get infected, clear the virus quickly, and do not transmit it to other individuals) [5, 27–30]. When many reservoir, or competent, host species occupy the same territory, an amplification of hantavirus prevalence occurs because of the hosts’ inability to clear the virus quickly, leading more virions to be continuously and actively shed in the environment [5, 28–30]. Dilution effect may occur when non-competent hosts occupy the same region as competent host species. The transmission of hantavirus to non-competent hosts decreases the amount of active hantavirus in the area, which leads to a dilution effect [5, 29, 30]. Several studies have attempted to decipher dilution/amplification effects on the overall hantavirus seroprevalence [31, 32]. Clay et al. [31] suggested that high species diversity at a site could affect SNV prevalence by reducing the number of deer mice; while Milholland et al. (2018) [25] found that higher species richness does not dilute SNV seroprevalence. The study also showed that phylogenetically diverse rodents have the potential to maintain SNV in the environment [25]. Additional studies have found multiple heteromyid species seropositive for SNV, though rodents in this taxonomic group were traditionally considered dilution agents [33, 34]. These findings call for further exploration of hantavirus host competence through genetic testing.

Since the initial outbreak, New Mexico (NM) continues to lead the USA in the number of HCPS cases, with 129 cases occurring between 1975 and 2023 and mortality rate of 43% [32]. These cases have primarily occurred in the north and northwestern regions of the state, with 97 of the 129 cases occurring in McKinley (N = 59), San Juan (N = 14), Taos (N = 14), and Cibola (N = 10) counties [35]. The reason for the persistent high incidence of HCPS cases in the four-corner region remains elusive. However, it was observed that the prevalence of SNV in deer mice showed a positive correlation with the abundance of deer mice, a variable that exhibited local and regional variations influenced by factors such as vegetation cover and climate [36]. In the neighboring states to eastern New Mexico, data from the Center for Disease Control (CDC) indicated 8 and 49 HCPS cases reported between 1993 and 2021 in Oklahoma and Texas, respectively [37]. Specifically, within the period from 1993 to 2013, 29 HCPS cases were documented in the three Texas State Health Service Regions bordering New Mexico, with 23 of these cases occurring in the northwest region of Texas [38]. Due to the lack of reported HCPS cases, eastern NM has been overlooked in hantavirus surveillance efforts, despite the region’s economy relying heavily on crop farming, dairy production, and livestock raising, all of which are deemed to increase human-rodent interactions [39]. A recent study conducted in eastern NM found multiple rodent species positive for SNV-specific antibodies, suggesting that hantaviruses are naturally present in this region [40]. However, the presence of SNV has not yet been genetically confirmed. Studies conducted in Bailey, Deaf Smith, and Loving Counties of bordering west Texas found rodents positive for SNV-specific antibodies and multiple cases of HCPS cases have occurred in Gaines and Deaf Smith Counties [34, 41, 42]. The goal of this study was to assess the prevalence of SNV in rodent assemblages across a range of habitats and disturbance levels in eastern New Mexico using RT-qPCR on collected tissues. Due to minimal surveillance conducted in this region, we sought to identify which rodent species and areas might pose potential risk to human health. We also evaluated whether biotic factors (e.g., vegetation cover and rodent community composition) and abiotic factors (e.g., day of year) play important roles in the overall hantavirus prevalence (i.e., % infected individuals per site). Finally, we also aimed to evaluate whether or not potential amplification or dilution effects were occurring within our study sites.

## Materials and methods

Procedures for field rodent collection (i.e., trapping and collecting tissue samples) were conducted under the provision and permits issued by the New Mexico State University Institutional Animal Care and Use Committee (IACUC) (2019–016) and New Mexico Department of Game and Fish permits (authorization #3621) issued to I. Mali.

From March 2020 to May 2021, we surveyed a total of 20 sites (Table 1) across 8 counties in east New Mexico. Each study site was at least ~0.65 km^2^ and the distance between sites was > 20 km. Site conditions ranged from pristine (i.e., protected wildlife areas) to highly disturbed (i.e., recently harvested agricultural fields). Trapping occurred during nights with the least amount of moon illumination [43]. At each site, 500 Sherman traps (H.B. Sherman Traps, Inc., Tallahassee, FL, USA) were set at dusk in curvilinear transect lines and baited with a mixture of oatmeal, peanut butter, and vanilla extract. The traps remained in the field for three consecutive nights and were checked for captures each morning. Traps remained closed during the day and were reopened at dusk. Rodent captures were humanely euthanized using a double-kill method—overdose of isoflurane with cervical dislocation [44]. Upon euthanasia, necropsies were conducted, and standard measurements recorded (total length, tail length, hindfoot length, ear length, and weight). Between captures, we disinfected all equipment (i.e., dissecting trays, scalpels, leather field gloves etc.) with 90% ethanol and hydrogen peroxide and used new sterile laboratory grade gloves when harvesting organs. Lungs and blood were collected from every capture. Lung tissues were preserved in 1.5 ml microcentrifuge tubes filled with RNAlater (Invitrogen, Waltham, MA, USA, Thermo Fisher Scientific, Waltham, MA, USA). Blood was collected from the heart and thoracic cavities onto Nobuto strips and into 1.5 ml microcentrifuge tubes, then the tubes were immediately centrifuged to separate serum from other blood components. Lung and blood tissues were stored at -70 °C until laboratory analysis. Rodents were identified in the field by visual inspection, taking into account key morphological features (e.g., hair coloration) and standard measurements (e.g., tail length, ear length, etc.). For specimens where visual inspection was inconclusive, which was the case with a few adult and all juvenile *Peromyscus* spp., we used genetic confirmation by sequencing mitochondrial *Cytb* gene [30].

**Table 1:** A summary of 20 sites surveyed for rodents from March 2020 to May 2021 across east New Mexico. The sites were surveyed over three-consecutive nights, and the second date of survey is depicted in the table. Specific coordinates are not provided in the table due to landowner privacy. Habitat type was determined by combining the results of vegetation surveys and evaluating dominant vegetation types. Habitats that were used to grow crops, graze livestock or heavily altered by human activity in other ways were classified as disturbed.

Name	County of Survey	Survey Date	Habitat Type
Site 1	Roosevelt	18 March 2020	Disturbed
Site 2	Curry	29 March 2020	Disturbed
Site 3	Chaves	19 April 2020	Grassland
Site 4	Roosevelt	26 April 2020	Shrubland
Site 5	Curry	15 May 2020	Shrubland
Site 6	Lea	16 June 2020	Grassland
Site 7	Lea	26 June 2020	Disturbed
Site 8	Eddy	16 July 2020	Shrubland
Site 9	Chaves	23 July 2020	Grassland
Site 10	Roosevelt	15 August 2020	Grassland
Site 11	Eddy	22 August 2020	Shrubland
Site 12	Quay	13 September 2020	Disturbed
Site 13	De Baca	20 September 2020	Disturbed
Site 14	Quay	11 October 2020	Shrubland
Site 15	Guadalupe	18 October 2020	Shrubland
Site 16	Chaves	8 March 2021	Shrubland
Site 17	Roosevelt	13 March 2021	Shrubland
Site 18	Quay	8 April 2021	Shrubland
Site 19	Eddy	13 April 2021	Grassland
Site 20	Eddy	8 May 2021	Shrubland

Additionally, vegetation surveys were conducted at each site using the Daubenmire method [45]. From the vegetation survey data, species composition, overall frequency of vegetation types, and percent cover of each vegetation type was identified and calculated. Then, survey sites were split into three habitat type categories based on vegetation surveys and observation of habitat disturbance: (1) disturbed; land noticeably disturbed by crop or livestock activity, (2) grassland; land dominated by grasses and not noticeably disturbed, and (3) shrubland; land dominated by shrubs and not noticeably disturbed.

### Laboratory analysis

As a preliminary assessment, indirect enzyme-linked immunoassays (ELISAs) were conducted for each rodent using blood. All samples were run in duplicates and both duplicates had to generate positive results in order for rodent to be considered positive for SNV-specific antibodies. Each test contained a negative and positive control. The assays were conducted under a Biosafety Level Two hood (BSL-2), using aseptic technique. The protocol followed for the assays was developed by Schountz et al. [46]. Any sample with the optical density (OD) of 0.2 units greater than the negative control was considered positive. The results were interpreted qualitatively, as the binding affinity of antibodies was measured.

RNA was extracted from frozen lung tissues using QIAmp^®^ Viral RNA Mini Kit (Qiagen, Hilden, Germany). RNA extraction followed the manufacturer’s instruction, with slight modifications to optimize RNA yield. Approximately 20–30 mg of frozen lung tissue was sectioned on dry ice and placed into a preloaded bead beater tube containing 1.0g each of 1.0 mm (catalog number 1107911zn; BioSpec, Bartlesville, OK, USA) and 2.3 mm Zirconia beads (catalog number 1107912zxy0; BioSpec, Bartlesville, OK, USA) in 600–800 μl of AVL buffer. Between each tissue, forceps were cleaned using 90% ethanol. Each tissue blade was only used once and then disposed of. Often throughout the RNA extraction process, the areas and equipment were cleaned with 90% ethanol, followed by RNase-Away to prevent any cross-contamination. The lung tissue was homogenized using bead beater machines. Samples were homogenized using either a BeadBugTM 6 Microtube Homogenizer (Benchmark Scientific, Sayreville, NJ, USA) for 2 cycles at 4,300 rpm for 30 seconds with 1 minute intervals between cycles, or a Mini-Beadbeater 16 for 2 cycles for 45 seconds with 1 minute intervals between cycles. Homogenates were centrifuged for 7 minutes at 7,000 rpms, then the clear lysate was pipetted into a 1.7 ml microcentrifuge tube, to which RNA carrier was added. The remaining isolation was performed as per manufacturer’s instruction using 30–50 μl of nuclease free water. Reverse transcription (cDNA synthesis) was conducted using SuperScript II (Invitrogen, Waltham, MA, USA, Thermo Fisher Scientific, Waltham, MA, USA). The cDNA synthesis was performed in 1.5 ml microcentrifuge tubes, using 5 μL of extracted RNA with 1 μl of random primers, 1 μl of dNTP, 5 μl of RT-qPCR grade H_2_O, 4 μl of 5x First Strand Buffer, 2 μl 0.1 M DTT, and 1 μl of RNAseOUT per each sample. The microcentrifuge tubes were incubated at 65°C for 5 minutes, iced briefly, then incubated for 10 minutes at room temperature. The cDNA was synthesized at 42°C for 50 minutes, then the reaction was terminated by incubating for 15 minutes at 70°C.

Polymerase chain reactions (PCR) analyses were run on the samples using Taqman Fast Advanced Master Mix. The primers and sequence information can be found in Goodfellow et al. [26]. The PCR machine used was a QuantStudio 5 Real-Time PCR Machine (Applied Biosystems, Waltham, MA, USA). Each PCR plate contained standard curve wells of 10^−1^ (^~^1,000 ng/μl) to 10^−10^ (^~^100 ng/μl) viral plasmid, a positive viral control (isolated RNA from SNV-infected Vero E6 cells), a positive rat/mouse control (from a sample in which SNV was detected through prior RT-qPCR), a negative rat/mouse control, and non-template control (which contains master mix, but no sample) wells in addition to the samples. Each control and sample were run in duplicates. The PCR reactions were carried out in 20 μl volumes, with each well containing 10 μl of Taqman Fast Master mix, 1 μl of probe, 5 μl of ddH_2_O, 1 μl of primer (half forward and half reverse primer) and 2 μl of each sample cDNA. The PCR was set up for 40 cycles with volume size set to 20 μl, with run mode set to fast. The holding stage was set to 95°C for 20 seconds, the PCR stage at 95°C for 1 second, and an annealing temperature at 52°C for 20 seconds. After PCR concluded, the limit of detection was set above each negative control and non-template controls, and any sample that amplified a cycle threshold (CT-value) of less than 40 was considered positive.

### Statistical analysis

Statistical analyses were conducted using generalized-linear models (GLM) in program R [47]. Based on the results of RT-qPCR tests, each rodent was assigned a value “0” if it tested negative and “1” if it amplified SNV specific primers (i.e., tested positive). The proportion of SNV positive rodents per site was then treated as a response variable, while eight explanatory variables included: 1) day of year (DOY), 2) % vegetation cover, 3) habitat type (i.e., grassland, croplands, disturbed land), 4) Simpson’s Diversity Index (SDI) [48] 5), total abundance index (i.e., total number of rodents captured per site), 6) sex (% male), 7) presence/absence of *P*. *maniculatus*, and 8) relative abundance of *P*. *maniculatus* (Table 2). Presence/absence of *P*. *maniculatus* was included in the models as we hypothesized that this reservoir host may contribute to the persistence of SNV in the environment and higher chance of infection events. In addition, the relative abundance of *P*. *maniculatus* at each site was included in the statistical model to test whether higher *P*. *maniculatus* abundance led to higher SNV prevalence at each site. Total abundance index was used to assess whether higher rodent abundance leads to higher SNV prevalence, an inference to higher contact rates among the members of rodent assemblages. The proportion of males was used to evaluate whether SNV disproportionally affects one sex, based on the speculation that males will show higher SNV prevalence due to their naturally aggressive behavior [49]. Overall vegetation cover served as a proxy for food and cover, with the speculation that less cover would lead to greater competition for resources and consequently higher SNV prevalence. We hypothesized that disturbed habitats would have higher prevalence of SNV infected rodents due to the main reservoir host, *P*. *maniculatus*, tendency to occupy suboptimal habitats and shed the virus into the environment [21, 50]. Prior to analyses, all continuous parameters were standardized to have a mean of zero and a standard deviation of one. All explanatory variables were checked for collinearity using Pearson’s correlation coefficient and excluded if they were highly correlated (≥ 0.7).

**Table 2:** The mean, standard deviation (SD), minimum, and maximum values of the continuous and frequency for categorical explanatory variables used in generalized linear models to estimate the prevalence of Sin Nombre Virus (SNV) in rodents of east New Mexico.

Explanatory Variables	Type	Summary
Simpson’s Diversity Index (SDI)	Continuous	Mean ± SD: 0.6483 ± 0.2001
		Range: 0.131–0.8874
Vegetation Cover (VegCover)	Continuous	Mean ± SD: 0.3930 ± 0.2660
		Range: 0.1000%– 1.00
Day of Year (DOY)	Continuous	Mean ± SD: 165.1750 ± 75.4360
		Range: 66.5–291.5
Total Abundance Index	Continuous	Mean ± SD: 36.9000 ±18.8928
		Range: 15–79
Males Relative Abundance (MaleRA)	Continuous	Mean ± SD: 0.4277 ± 0.0958
		Range: 0.2593–0.6222
*Peromyscus maniculatus* Relative Abundance (manicRA)	Continuous	Mean ± SD: 0.5744 ± 0.3112
		Range: 0.0253–0.9333
Habitat Type (HabType)	Categorical	5 crop/overgrazed sites
		5 grassland sites
		10 shrubland sites
Presence of *Peromyscus maniculatus* (manicPres)	Categorical	9 absent
		11 present

To account for overdispersion, we used GLM with quasi-binomial distribution [51]. As quasi-binomial models do not generate Akaike Information Criterion (AIC) values, we used *drop1* function to select the best fit model [51]. This command evaluates the effect of dropping each explanatory variable within a full model, and in turn each time applies an analysis of deviance test [51]. The variable with the highest p-value was removed and we repeated the process until all explanatory variables were significant (p > 0.05). We additionally performed chi-square tests of independence to assess whether there was a difference in SNV prevalence between sexes (i.e., male, female, and juvenile) and rodent families (i.e., Cricetidae vs. Heteromyidae).

## Results

**Fig 1:**
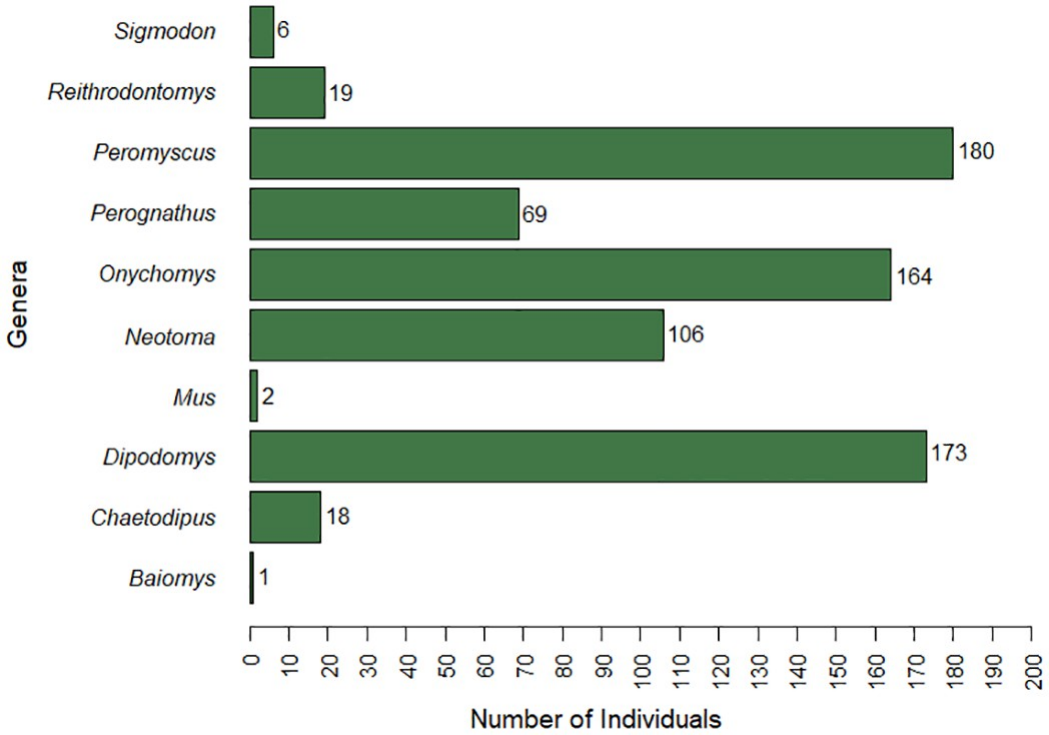
A bar graph depicting the number of individuals captured per genus during rodent surveys in east New Mexico from March 2020 to May 2021. A total of 738 rodents were captured from three families (64% Cricetidae, 35% Heteromyidae, and <1% Muridae), across 10 genera, and 23 species.

**Fig 2:**
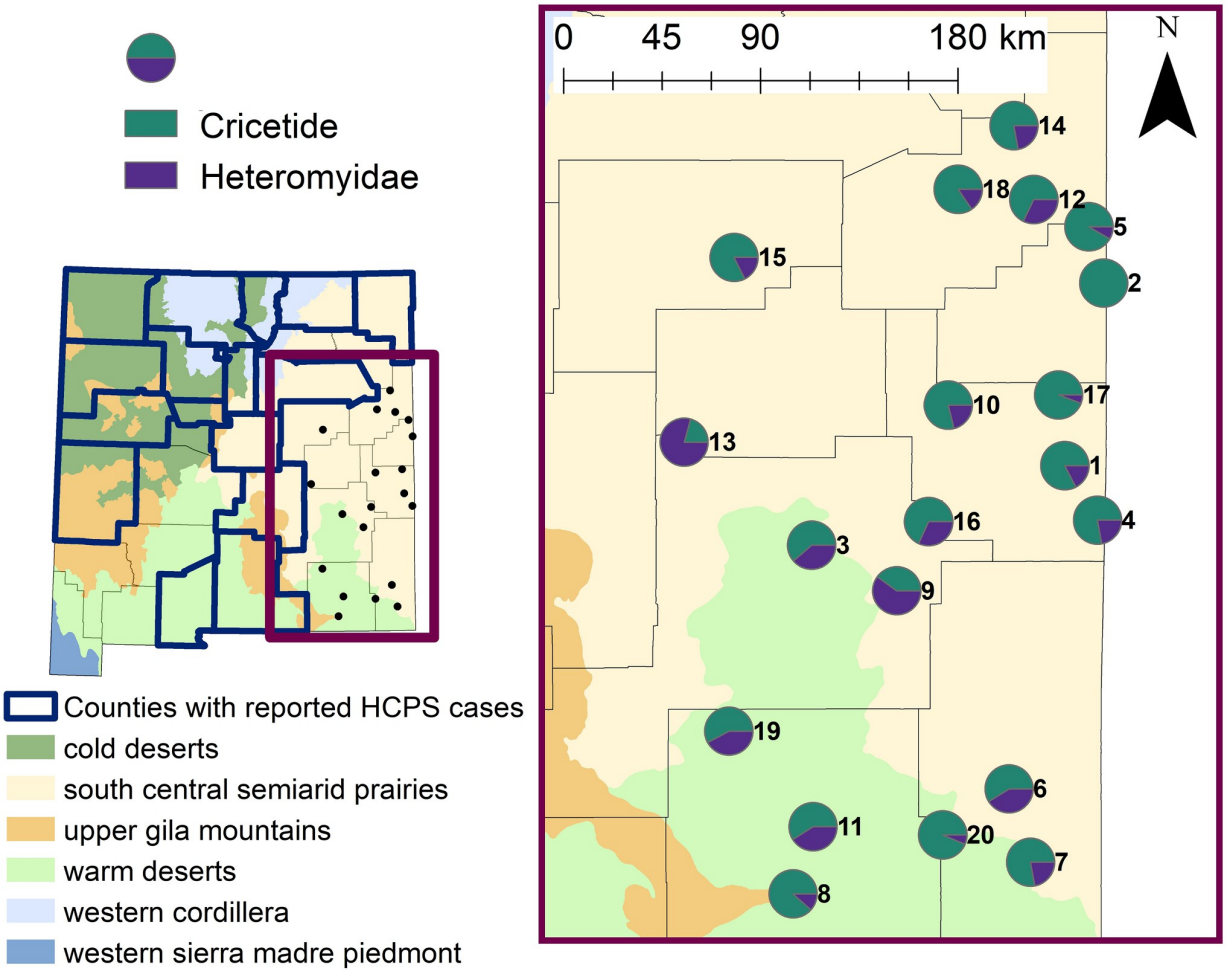
Map of the state of New Mexico (left) highlighting counties that have reported cases of hantavirus cardiopulmonary syndrome based on the New Mexico Department of Health data in relation to 20 sites surveyed for rodents across east New Mexico in 2020 and 2021. Map of east New Mexico (right) including the relative proportion of Cricetid and Heteromyid rodents captured across the 20 survey sites. Site numbers correspond to Table 1. Level II Ecoregion GIS data was sourced from the freely available United States Environmental Protection Agency—Ecoregion database (https://www.epa.gov/eco-research/ecoregions-north-america).

Across 20 survey sites and 30,000 trap nights, a total of 738 individual rodents were captured (Fig 1; S1 Table). In total, 23 species of rodents were identified, comprising 10 genera, with majority of captures belonging to family Cricetidae (64%), followed by Heteromyidae (35%), and <1% belonged to Muridae (Fig 2). Capture per unit effort ranged from 1% (i.e., 15 captures/1,500 trap nights) to 5.2%. Species richness ranged from 2 to 9 per site, while species diversity index (i.e., SDI) ranged from 0.13 to 0.89. Six species of *Peromyscus* were captured across 17 sites, of which *P*. *maniculatus* was caught at 11 sites. Overall, the most abundantly captured species were *Onychomys leucogaster* (N = 164), *P*. *maniculatus* (N = 113), and *Dipodomys merriami* (N = 108).

Out of the 738 rodent samples, only 51 (6.9%) were found to be seropositive for SNV antibodies among 8 species (Table 3). Among seropositive individuals, 61% were Cricetid rodents (31/51) and 39% were Heteromyids (20/51). Seroprevalence was similar between males and females (χ^2^ = 0.05; df = 1; p = 0.83). Overall, there was also no difference in seropositivity rate between the two families (χ^2^ = 0.36; df = 1; p = 0.55). Sixteen study sites had seropositive individuals. Among those, the proportion of seropositive individuals at each site ranged 1.9% to 33.3%, with a mean of 8.9%.

**Table 3:** A summary of the rodent species that tested positive for SNV specific antibodies through enzyme-linked immunosorbent assays (ELISAs) on the blood samples collected from the samples during this study, including the number of seropositive individuals (“+” sign) per sex as well as the overall prevalence. Species that did not test seropositive were excluded from this table. Rodents were surveyed from March 2020 to May 2021 across 20 sites in east New Mexico.

Genus	Species	Male (+)	Female (+)	Juvenile (+)	+/Total Captured	Seropositivity Rate
*Neotoma*	*leucodon*	3	2	0	5/41	12.20%
*Neotoma*	*micropus*	4	6	1	11/65	16.92%
*Onychomys*	*leucogaster*	2	1	0	3/164	1.83%
*Peromyscus*	*leucopus*	0	1	0	1/32	3.13%
*Peromyscus*	*maniculatus*	9	1	0	10/113	8.85%
*Reithrodontomys*	*megalotis*	0	1	0	1/8	12.50%
*Dipodomys*	*merriami*	2	9	0	11/106	10.38%
*Dipodomys*	*ordii*	4	5	0	9/64	14.06%

**Fig 3:**
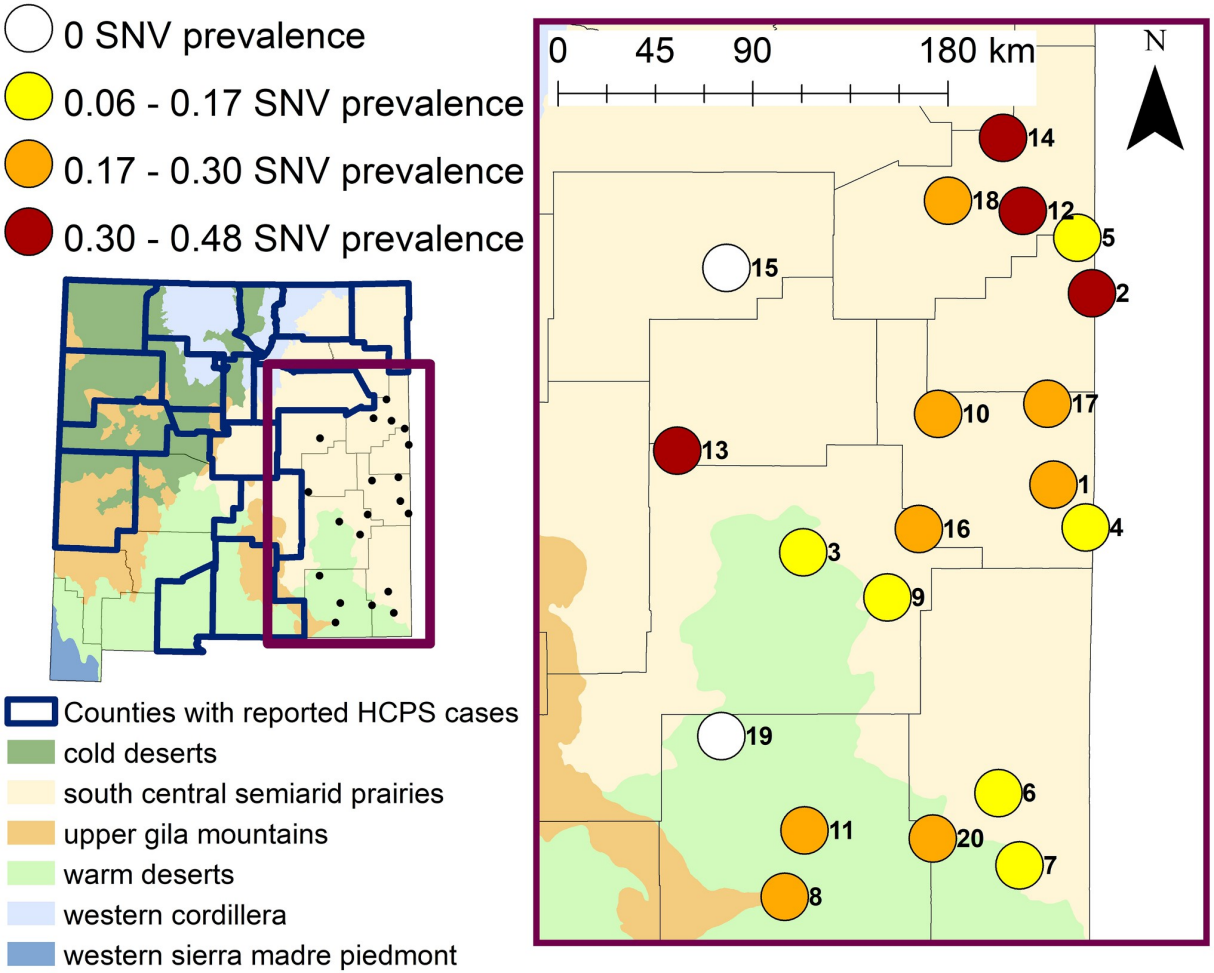
Map of the state of New Mexico (left) highlighting counties that have reported cases of hantavirus cardiopulmonary syndrome based on the New Mexico Department of Health data in relation to 20 sites surveyed for rodents across east New Mexico in 2020 and 2021. Map of east New Mexico (right) displaying the SNV positivity rate of rodents captured at each survey site based on results from the RT-qPCR analyses using SNV specific primers. Site numbers correspond to Table 1. Level II Ecoregion GIS data was sourced from the freely available United States Environmental Protection Agency—Ecoregion database (https://www.epa.gov/eco-research/ecoregions-north-america).

Through RT-qPCR, a total of 167 samples (22.6%) across 16 species were positive for SNV (Table 4). Mean Ct-values per species ranged from 34.8 (*Dipodomys spectabilis*) to 39 (*Mus musculus*). *Peromyscus maniculatus* samples had a mean Ct-value of 37, which is also the overall mean value per species (i.e., mean of means per species). For positive RT-qPCR samples, the mean Ct-values did not differ drastically between seropositive and seronegative Cricetid or Hereomyid rodents (i.e., difference of less than 1.15). Prevalence between Cricetid and Heteromyid captures in this study was similar overall (χ^2^ = 0.73; df = 1; p = 0.39) and per site (S2 Table), with the exception of site no. 10 where Heteromyids had higher SNV prevalence than Cricetids (χ^2^ = 5.24; df = 1; p = 0.02). Interestingly, this site also had the highest proportion of Heteromyid rodents (Fig 2) and one of the highest prevalence rates (Fig 3). SNV infection rate was 24.2% (80/330) and 21.7% (73/337) for males and females, respectively. There was no statistically significant difference in SNV prevalence between sexes per site (p > 0.05; S3 Table) and overall (χ^2^ = 0.63; df = 1, p = 0.43). Eighteen sites had at least one individual positive for SNV (Fig 3). The proportion of positive individuals at the 18 sites ranged from 5.6% to 47.5%, with a mean of 22.8% (Fig 3). Site no. 12, which was a disturbed site located in Quay County, had both the highest number of positive individuals and the greatest proportion of positive individuals. At this site, SDI was 0.8521 and *P*. *maniculatus* constituted only 4 of 59 total captures. The best fit generalized linear model indicated that of all covariates considered, only habitat type significantly influenced the overall SNV prevalence within rodent assemblages (Table 5). Specifically, the proportion of SNV infected rodents was significantly lower in grassland (p = 0.005) and shrubland (p = 0.039) habitats than in disturbed habitats.

**Table 4:** A summary of RT-qPCR results tested for Sin Nombre Virus (SNV) across all rodents captured during 2020 and 2021 surveys in east New Mexico. Each species is split according to the sex category. “+” represents the number of positive individuals within the category.

Family	Genus	Species	Male (+/total)	Female (+/total)	Juvenile (+/total)	Total Captured	Positivity Rate
Cricetidae	*Baiomys*	*taylori*	-	0/1	-	1	0.00%
Cricetidae	*Neotoma*	*leucodon*	2/16	6/23	0/2	41	19.51%
Cricetidae	*Neotoma*	*micropus*	4/17	3/34	3/14	65	15.38%
Cricetidae	*Onychomys*	*leucogaster*	23/87	12/67	3/10	164	23.17%
Cricetidae	*Peromyscus*	*eremicus*	0/3	1/6	1/1	10	20.00%
Cricetidae	*Peromyscus*	*leucopus*	6/16	2/13	1/3	32	28.13%
Cricetidae	*Peromyscus*	*maniculatus*	21/52	13/43	3/18	113	32.74%
Cricetidae	*Peromyscus*	*nasutus*	0/1	0/1	0/3	5	0.00%
Cricetidae	*Peromyscus*	*pectoralis*	2/5	1/3	0/1	9	33.33%
Cricetidae	*Peromyscus*	*truei*	0/6	1/4	0/1	11	9.09%
Cricetidae	*Reithrodontomys*	*megalotis*	0/3	2/5	-	8	25.00%
Cricetidae	*Reithrodontomys*	*montanus*	1/4	1/4	0/3	11	18.18%
Cricetidae	*Sigmodon*	*hispidus*	0/3	0/3	-	6	0.00%
**Total**			**59/213**	**42/207**	**11/56**	**476**	**23.53%**
Heteromyidae	*Chaetodipus*	*eremicus*	0/1	-	-	1	0.00%
Heteromyidae	*Chaetodipus*	*hispidus*	1/3	3/9	2/4	16	37.50%
Heteromyidae	*Chaetodipus*	*intermedius*	0/1	-	-	1	0.00%
Heteromyidae	*Dipodomys*	*merriami*	6/51	8/50	1/5	106	14.15%
Heteromyidae	*Dipodomys*	*ordii*	7/27	7/33	0/4	64	21.88%
Heteromyidae	*Dipodomys*	*spectabilis*	1/1	1/2	-	3	66.67%
Heteromyidae	*Perognathus*	*flavus*	5/30	12/34	0/1	65	26.15%
Heteromyidae	*Perognathus*	*flavenscens*	-	-	0/1	1	0.00%
Heteromyidae	*Perognathus*	*merriami*	0/1	0/2	-	3	0.00%
**Total**			**20/115**	**31/130**	**3/15**	**260**	**20.77%**
Muridae	*Mus*	*musculus*	1/2	-	-	2	50.00%
**Total**			**1/2**	**-**	**-**	**2**	**50.00%**

**Table 5:** Output of the top model that best explained the proportion of Sin Nombre Virus (SNV) positive rodents in east New Mexico based on results from the RT-qPCR analyses using SNV specific primers. Parameter estimates and standard errors are reported on the logit scale. Disturbed land was the reference category for the habitat type.

Parameter	Estimate	Standard Error	P-Value
Intercept	-0.6852	0.2168	<0.01
Grassland	-1.4379	0.4522	0.005
Shrubland	-0.6111	0.2736	0.039

Comparing the results of the ELISA and RT-qPCR testing, there were only 15 samples that were mutually positive (i.e., positive through both analyses). These samples belonged to *Dipodomys ordii* (N = 2), *Neotoma leucodon* (N = 2), *Neotoma micropus* (N = 3), *Onychomys leucogaster* (N = 1), and *Peromyscus maniculatus* (N = 7). SNV was detected only through RT-qPCR in three Heteromyid rodents (*Chaetodipus hispidus*, *Dipodomys spectabilis*, *Perognathus flavus*), four Cricetid species (*Peromyscus eremicus*, *Peromyscus pectoralis*, *Peromyscus truei*, *Reithrodontomys montanus*), and in *Mus musculus* (S1 Fig). Across all species, the prevalence was higher through RT-qPCR results than through ELISA, with one exception (*Neotoma micropus*; S1 Fig). For the most competent host, *Peromyscus maniculatus*, three individuals that seroconverted did not test positive for the virus through RT-qPCR while 30 individuals that were genetically positive did not seroconvert.

## Discussion

To date, this project represents the most comprehensive hantavirus surveillance effort conducted in eastern NM, where there are no reported HCPS cases. We built upon the preliminary study of Curtis et al. [40] and increased trap effort, covered a larger geographical area, and used additional and more sensitive laboratory techniques (i.e., RT-qPCR) to assess the presence of SNV. Similarly to Curtis et al. [40] we confirm that SNV is widespread across rodent assemblages in eastern New Mexico. Of 20 survey sites, SNV was not detected at only two sites—one in Eddy County and one in Guadalupe County (Fig 3).

Among the most abundant species caught, *P*. *maniculatus* had the highest SNV prevalence (33%), followed by *O*. *leucogaster* (20%) and *D*. *merriami* (11%), but an additional 13 species also tested positive for SNV. SNV was genetically detected in six previously unreported host species: *Onychomys leucogaster* (Northern grasshopper mouse), *Dipodomys merriami* (Merriam’s kangaroo rat), *D*. *ordii* (Ord’s kangaroo rat), *D*. *spectabilis* (Banner-tailed kangaroo rat), *Perognathus flavus* (Silky pocket mouse) and *Chaetodipus hispidus* (Hispid pocket mouse). All have been reported seropositive in the literature, but have not been further genetically confirmed [e.g., 30, 40, 52–54]. We also detected SNV in non-native *M*. *musculus* (House mouse), a known host to multiple infectious hantaviruses including Puumala-like viruses, Seoul virus, and SNV [18, 26, 55]. The presence of SNV in multiple rodent species stresses the importance of testing entire rodent assemblages when evaluating the SNV infection risk and hantavirus dynamics across landscapes, as we detected hantavirus in both known competent and traditionally considered non-competent hosts.

In our study, SNV prevalence was significantly lower in relatively undisturbed grassland and shrubland habitat, when compared to disturbed habitat types. Generalist species, such as *P*. *maniculatus*, are known to occupy suboptimal or disturbed habitats, thus potentially increasing the amount of hantavirus being shed in the environment [21, 50, 56, 57]. In our study, there were more *P*. *maniculatus* captured at disturbed sites (N = 79/113; 70%), than at undisturbed sites (N = 34/113; 30%). Overall, this study brings into question many assumptions about the SNV prevalence. For example, there were no significant differences in SNV prevalence between males and females; therefore, the hypotheses that higher aggression increases prevalence in males was not supported. The hypothesis that total abundance index, proxy to higher rodent abundance, could potentially lead to a higher SNV prevalence was also unsupported. It was also hypothesized that an increase in species diversity would dilute the prevalence of SNV because, at least traditionally, only *P*. *maniculatus* was considered a highly competent host, although this has been disputed recently [26, 58]. One of the reasons for the lack of SDI effect on the overall prevalence could be because lower SDI in our study did not necessarily equate to high abundance of a highly competent host. While some of the study sites with low SDI consisted mainly of *P*. *maniculatus* (e.g., Site 2, SDI = 0.131, SNV prevalence = 40%), others consisted mainly of species not considered competent hosts such as *O*. *leucogaster* (e.g., Site 4, SDI = 0.354, SNV prevalence = 11%). Luis et al. [29] suggested that amplification/dilution can occur simultaneously based on species competence for the virus. However, many species in our study tested positive for SNV, clouding the ability to parse species competence and therefore dilution/amplification effects. We suspect that many rodent species may represent competent hosts, which suggests amplification effect is a more commonly occurring phenomenon.

The discrepancies in the results between the two laboratory approaches (ELISA vs qPCR) are perplexing. While the mechanisms of infectivity and immune response timeline for known reservoir species such as *Peromyscus maniculatus* is well-understood [17–20], there remains a significant lack of knowledge regarding these mechanisms in many other species. As a result, we can only speculate on the reasons for the observed significant variations. It is possible that rodents in our study were in different stages of infection or that potential cross-reactivity of a hantavirus closely related to SNV may have occurred [45, 59–61]. It is also possible that some of our ELISA samples showed negative results due to diminishing antibody response or sample degradation. Furthermore, it is important to acknowledge that complex rodent population dynamics might also influence the timeline of infection responses. Disparity between PCR and ELISA results for SNV have been reported previously, potentially due to time of sampling post-infection or differences between assay sensitivity [17].

Although many species in our study have tested positive for viral RNA, these results may not correlate to virus transmission. To comprehensively evaluate the potential risks of human infection, it is imperative that future research efforts focus on elucidating these mechanisms. Laboratory studies on the ability of other rodent species found positive for SNV to shed viral particles and infect others will be crucial in understanding SNV dynamics within rodent assemblages and its spillover to humans. It will be of particular interest to parse whether any Heteromyid rodents and Cricetid rodents previously unknown to science to contract SNV infection represent the dead-end hosts or actively contribute to the spread of the virus. It would also be beneficial for studies done in New Mexico to isolate and sequence the genomes of SNV positive rodents, to build potential phylogenetic relationship.

## Conclusions

This study genetically confirms that SNV exists in east New Mexico and across a wide spectrum of rodent species, including the known SNV reservoir *Peromyscus maniculatus*. However, the infectivity mechanisms and the capacity to release the virus into the environment for many other species identified in this study are currently unknown, making them subjects of future research interest. Our study indicated that the SNV prevalence is higher in disturbed habitats and although there have been no known-reported cases of HCPS in this region, the potential for human infection exists. Our assumption for the lack of HCPS cases in east New Mexico was the non-existent or low prevalence of SNV in rodent assemblages, but this was not supported in the current study. While this research provided a background knowledge for the prevalence of SNV in rodents, further studies on the human-dimention side of SNV vs. HCPS is needed. Because the first symptoms of HCPS are very similar to additional respiratory infections, it is possible that milder cases of HCPS, caused by strains with lower virulence, occur but are not being tested/reported. East New Mexico represents a rural part of the state where cattle ranching and oil and gas industry practices require people to spend a substantial amount of time outside in open areas with good ventilation which could decrease an infection risk. These are all speculations but important new avenues to consider when studying SNV in this portion of the state.

Nevertheless, scientists should focus on routine surveillance of entire rodent communities, as well as isolating and genotyping the viral strains from this region [62]. This study suggests that solely focusing hantavirus surveillance efforts on *Peromyscus* species, may significantly underestimate the amount of hosts that exist within a region. Conducting research on infectivity maintenance across a broad spectrum of species will be pivotal in predicting human infection risks. Identifying potential infection spillover areas, such as hot spot locations found in this study and evaluating which environmental variables most affect the presence and prevalence of SNV in this region can aid in epidemiological modelling [62]. Scientists can work directly with public health experts to disperse information about SNV, native reservoir hosts in New Mexico, and actions that they can take to reduce infection. Actions that members of the public can take include securing any potential rodent entry points in their house and clearing their yards of woodpiles and trash to avoid infestations [63–65]. When cleaning or conducting activities in a confined, poor-ventilated space, individuals should wear protective gloves and filtered masks [63–65] They may also spray disinfectants onto droppings to inactivate the virus prior to cleaning [63–65]. Although more research is warranted to evaluate the environmental and biological mechanisms behind the of lack human infection in this region, the results of this study indicate that actions should be taken to reduce or prevent infection outbreaks.

## Supporting information

S1 Fig Two bar graphs comparing the positivity results between the two detection methods, ELISA and RT-qPCR, used to test for the presence of SNV in rodent samples collected in east New Mexico from March 2020 to May 2021. Bar graph A compares the prevalence of SNV between species within the Heteromyidae family, while bar graph B compares the prevalence of SNV between species within the Cricetidae family. More samples were detected through RT-qPCR than ELISA, across both families.
(TIF)

S1 Table A table containing the rodent capture data for 738 samples captured between 2020–2021 in eastern New Mexico, USA. GPS coordinates were excluded from the table as some sites were on privately owned land. Results of ELISA testing are listed as "1" if the samples was seropositive for SNV and "0" if it was not. Similarly, results of qPCR are represented as "0" if SNV was not genetically detected through RT-qPCR, and "1" if it was.
(XLSX)

S2 Table A chi-square results comparing the differences in the number of SNV positive individuals based on qPCR between families (i.e., Heteromyidae and Cricetidae) for each site and for the overall captures between 2020–2021 in eastern New Mexico, USA. (XLSX)

S3 Table A chi-square results comparing the differences in the number of SNV positive individuals based on qPCR among sex classes (i.e., male, female, and juvenile) for each site and for the overall captures between 2020–2021 in eastern New Mexico, USA. (XLSX)
