# Supplementary figures and images for: Distribution and prevalence of Sin Nombre hantavirus in rodent species in eastern New Mexico
Source: PLoS One. 2024 Jan 18;19(1):e0296718. doi: 10.1371/journal.pone.0296718 (PMC10796054; doi:10.1371/journal.pone.0296718)

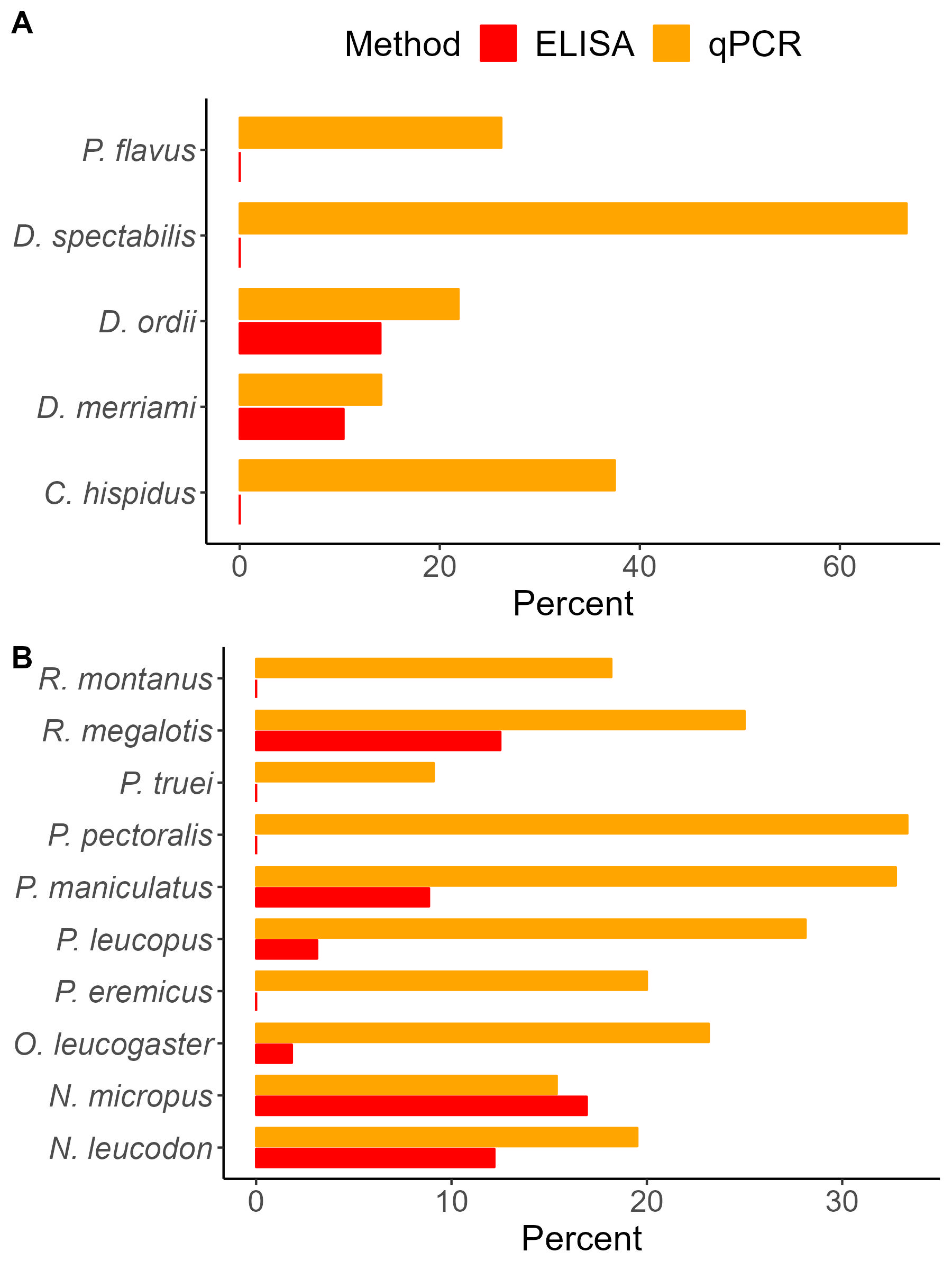

Supplement: S1 Fig — Bar graph A compares the prevalence of SNV between species within the Heteromyidae family, while bar graph B compares the prevalence of SNV between species within the Cricetidae family. More samples were detected through RT-qPCR than ELISA, across both families. (TIF) [file pone.0296718.s001.tif]
